# Supplementary material for: Optimising the yield from bronchoalveolar lavage on human participants in infectious disease immunology research
Source: Sci Rep. 2023 May 31;13:8859. doi: 10.1038/s41598-023-35723-2 (PMC10231287; doi:10.1038/s41598-023-35723-2)

Supplementary material

Optimising the yield from bronchoalveolar lavage on human participants in infectious disease immunology research

Jane Alexandra Shaw, Maynard Meiring, Devon Allies, Lauren Cruywagen, Tarryn-Lee Fisher, Kesheera Kasavan, Kelly Roos, Stefan Marc Botha, Candice MacDonald, Andriëtte M. Hiemstra, Donald Simon, Ilana van Rensburg, Marika Flinn, Ayanda Shabangu, Helena Kuivaniemi, Gerard Tromp, Stephanus T. Malherbe, Gerhard Walzl, Nelita du Plessis, & the SU IRG BAL Study Consortium.

Supplementary Tables

Supplementary Table S1. Interpretation of SARS-CoV-2 antibody test results according to vaccination status and type of antibody assay.

	Anti-N ab POSITIVE, unvaccinated	Anti-N ab POSITIVE, vaccinated	Anti-N ab NEGATIVE, vaccinated	Anti-N ab NEGATIVE, unvaccinated
**Anti-S ab POSITIVE**	Infection-induced immunity	Both infection-induced immunity and vaccine response, or waned vaccine response with breakthrough infection	Vaccine-induced immunity	Infection-induced immunity
**Anti-S ab NEGATIVE**	Infection-induced immunity	Waned vaccine response with breakthrough infection	No evidence of previous SARS-CoV-2 infection or vaccination	No evidence of previous SARS-CoV-2 infection or vaccination

Participants with serology results in the orange squares are included in the 'SARS-CoV-2 positive' subgroup; participants with serology results in the blue squares are included in the 'SARS-CoV-2 negative' subgroup.
Anti-N protein ab, anti-nucleocapsid protein antibody; Anti-S protein ab, anti-spike protein antibody; SARS-CoV-2.
Adapted from Hiemstra et al. Cascade Immune Mechanisms of Protection against Mycobacterium tuberculosis (IMPAc-TB): study protocol for the Household Contact Study in the Western Cape, South Africa. BMC Infect Dis. 2022 Apr;22(1):381 (main article reference 10), with permission of the authors.

Supplementary Table S2. Results of paired sample t-tests and quantile comparison tests for studies with participants who had bronchoalveolar lavage as both TB Early Treatment and TB End of Treatment.

			Robust t-test			Quantile comparison test						
Variable	Study	n	p-value	CI[low]	CI[high]	Quantile	Early Trt.	End of Trt.	Difference	CI[low]	CI[high]	p-value
Volume (ml)						0.25	80.584	71.203	9.38	-13.975	34.056	0.4
	MR1	15	0.249	-8.992	31.925	0.50	113.855	95.518	18.337	-4.671	38.858	0.12
						0.75	134.839	116.69	18.149	-16.414	41.232	0.243
						**0.25**	**56.467**	**102.258**	**-45.791**	**-69.388**	**-18.386**	**0**
	**MRM**	**6**	**0.043**	**-69.761**	**-1.573**	**0.50**	**75.898**	**114.081**	**-38.183**	**-65.388**	**-12.181**	**0.006**
						0.75	101.131	127.991	-26.86	-59.736	4.256	0.144
Total cells						0.25	1.84e+07	7.56e+06	1.08e+07	-1.32e+06	2.90e+07	0.084
	**MR1**	**14**	**0.025**	**3.31e+06**	**4.17e+07**	**0.50**	**3.94e+07**	**1.55e+07**	**2.39e+07**	**2.39e+06**	**4.45e+07**	**0.026**
						**0.75**	**6.73e+07**	**3.13e+07**	**3.60e+07**	**7.21e+06**	**6.07e+07**	**0.012**
						0.25	4.07e+06	1.38e+07	-9.72e+06	-3.32e+07	5.35e+06	0.138
	MRM	6	0.248	-4.21e+07	1.37e+07	0.50	1.27e+07	3.19e+07	-1.92e+07	-3.77e+07	1.05e+07	0.182
						0.75	2.73e+07	4.39e+07	-1.66e+07	-3.77e+07	6.23e+06	0.126
Viability						0.25	0.955	0.953	0.001	-0.014	0.031	0.928
	MR1	14	0.84	-0.018	0.022	0.50	0.961	0.967	-0.006	-0.017	0.011	0.461
						0.75	0.971	0.975	-0.004	-0.018	0.016	0.692
						0.25	0.945	0.958	-0.014	-0.06	0.023	0.631
	MRM	6	0.498	-0.041	0.023	0.50	0.969	0.97	-0.001	-0.033	0.014	0.585
						0.75	0.974	0.98	-0.006	-0.01	0.003	0.156

For quantile comparisons, the quantile estimator proposed by Harrell and Davis was used (HD estimator). MRM and MR1 are the studies in which these participants were enrolled.

Supplementary Table S3. Results of two-way ANOVA on Modified M-estimators of clinical groups with and without the effect of smoking considered. In the smoking groups, a significant P value denotes a group interaction where smoking significantly affected the outcome.

BAL Volume Yield:

Interaction	Group 1	Group 2	Estimate	p
Main effect	Community Control	Household Contact	-10.84	0.357
	Community Control	TB pre-treatment	25.73	0.357
	Community Control	TB early treatment	39.16	0.070
	Community Control	TB EOT	60.45	<0.001
	Household Contact	TB pre-treatment	36.57	0.357
	Household Contact	TB early treatment	50	0.070
	Household Contact	TB EOT	71.29	0.010
	TB pre-treatment	TB early treatment	13.43	0.357
	TB pre-treatment	TB EOT	34.72	0.357
	TB early treatment	TB EOT	21.29	0.356
Interaction with smoking	Community Control	Household Contact	1.298	0.357
	Community Control	TB pre-treatment	-22.33	0.357
	Community Control	TB early treatment	7.456	0.357
	Community Control	TB EOT	16.96	0.335
	Household Contact	TB pre-treatment	-23.63	0.357
	Household Contact	TB early treatment	6.158	0.357
	Household Contact	TB EOT	15.66	0.326
	TB pre-treatment	TB early treatment	29.79	0.357
	TB pre-treatment	TB EOT	39.29	0.357
	TB early treatment	TB EOT	9.505	0.357

BAL Cell Yield:

Interaction	Group 1	Group 2	Estimate	p
Main effect	Community Control	Household Contact	-1.209e+07	0.147
	Community Control	TB pre-treatment	-4.187e+07	<0.001
	Community Control	TB early treatment	-1.151e+07	0.300
	Community Control	TB EOT	3.728e+06	0.483
	Household Contact	TB pre-treatment	-2.978e+07	0.038
	Household Contact	TB early treatment	5.814e+05	0.485
	Household Contact	TB EOT	1.582e+07	0.217
	TB pre-treatment	TB early treatment	3.036e+07	0.038
	TB pre-treatment	TB EOT	4.56e+07	0.030
	TB early treatment	TB EOT	1.524e+07	0.300
Interaction with smoking	Community Control	Household Contact	6.328e+04	0.485
	Community Control	TB pre-treatment	2.07e+07	0.079
	Community Control	TB early treatment	5.466e+04	0.485
	Community Control	TB EOT	3.696e+07	0.037
	Household Contact	TB pre-treatment	2.064e+07	0.147
	Household Contact	TB early treatment	-8624	0.485
	Household Contact	TB EOT	3.69e+07	0.047
	TB pre-treatment	TB early treatment	-2.064e+07	0.197
	TB pre-treatment	TB EOT	1.626e+07	0.254
	TB early treatment	TB EOT	3.691e+07	0.074

BAL Concentration yield:

Interaction	Group 1	Group 2	Estimate	p
Main effect	Community Control	Household Contact	-6.772e+04	0.314
	Community Control	TB pre-treatment	-4.377e+05	<0.001
	Community Control	TB early treatment	-9.289e+04	0.250
	Community Control	TB EOT	-4.892e+04	0.456
	Household Contact	TB pre-treatment	-3.7e+05	<0.001
	Household Contact	TB early treatment	-2.517e+04	0.410
	Household Contact	TB EOT	1.88e+04	0.429
	TB pre-treatment	TB early treatment	3.448e+05	0.007
	TB pre-treatment	TB EOT	3.888e+05	0.010
	TB early treatment	TB EOT	4.397e+04	0.379
Interaction with smoking	Community Control	Household Contact	4.666e+04	0.355
	Community Control	TB pre-treatment	2.079e+05	0.072
	Community Control	TB early treatment	5.236e+04	0.429
	Community Control	TB EOT	2.693e+05	0.066
	Household Contact	TB pre-treatment	1.613e+05	0.163
	Household Contact	TB early treatment	5706	0.456
	Household Contact	TB EOT	2.226e+05	0.111
	TB pre-treatment	TB early treatment	-1.556e+05	0.217
	TB pre-treatment	TB EOT	6.134e+04	0.379
	TB early treatment	TB EOT	2.169e+05	0.144

Supplementary Figures

Supplementary Figure S1. Variability in BAL cell yields over time.

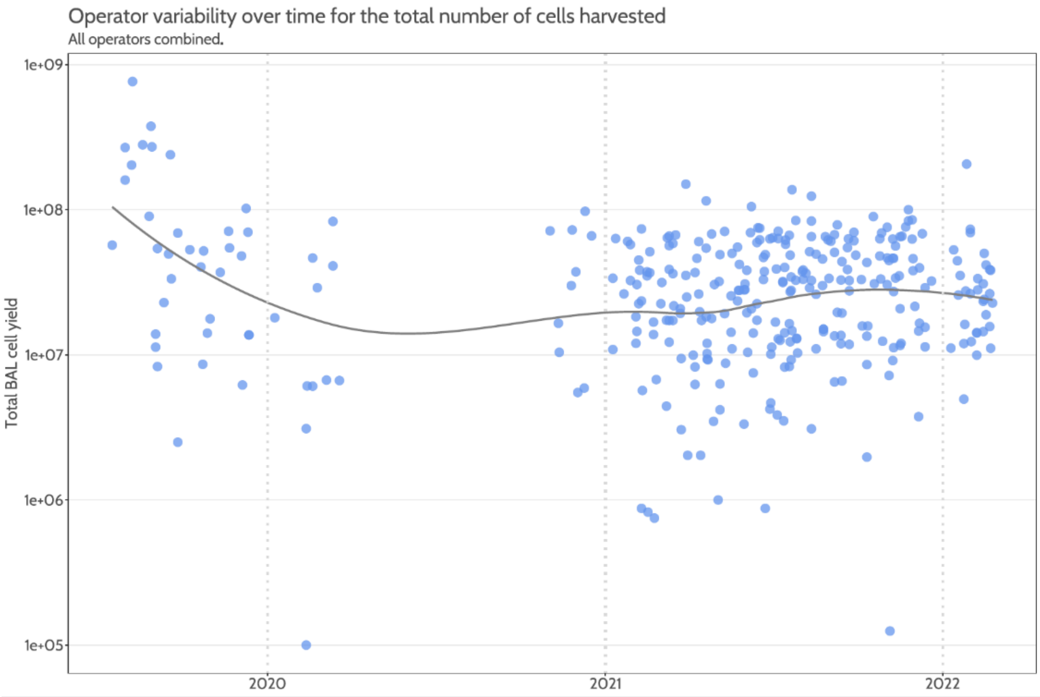

Panel A. There was no significant variability in the BAL cell yield over time (all parent studies and all operators combined). The apparent higher yields pre-2020 were not significant on analysis, but might be attributed to the initial recruitment of only participants with active TB disease, who yield higher cell counts than other clinical groups (as shown in the main article). The gap in recruitment is attributable to the national lockdown at the beginning of the COVID-19 pandemic.

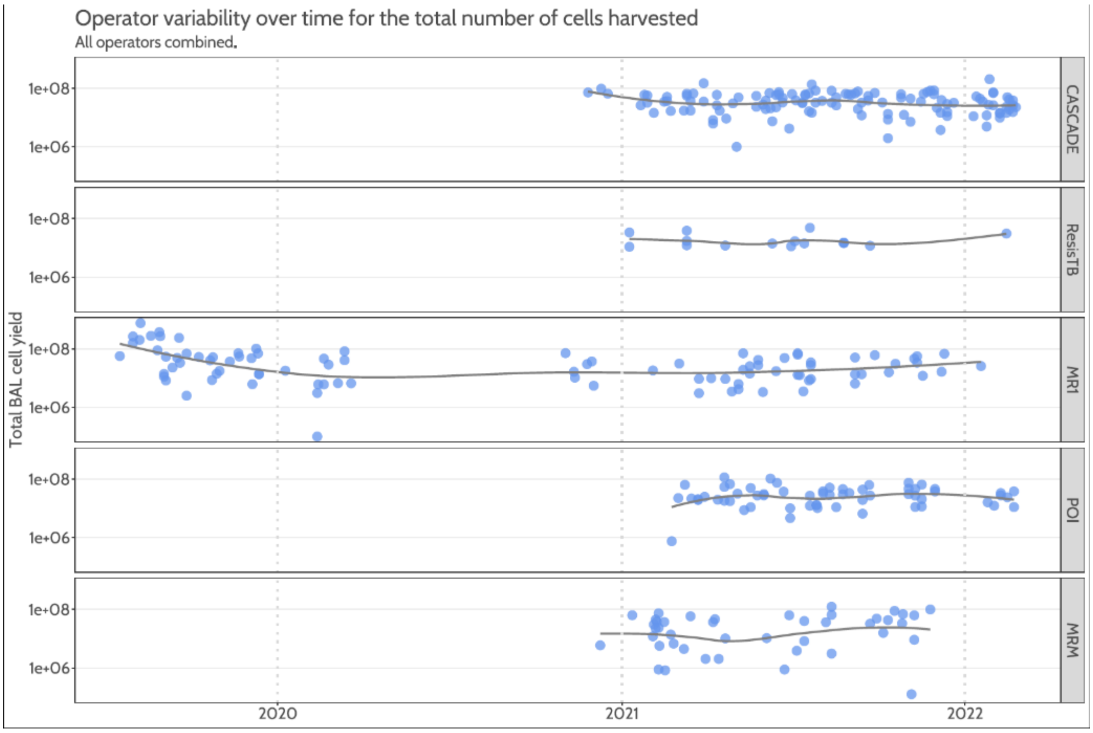

Panel B. There was no significant variability in the BAL cell yield over time when stratified by parent study.

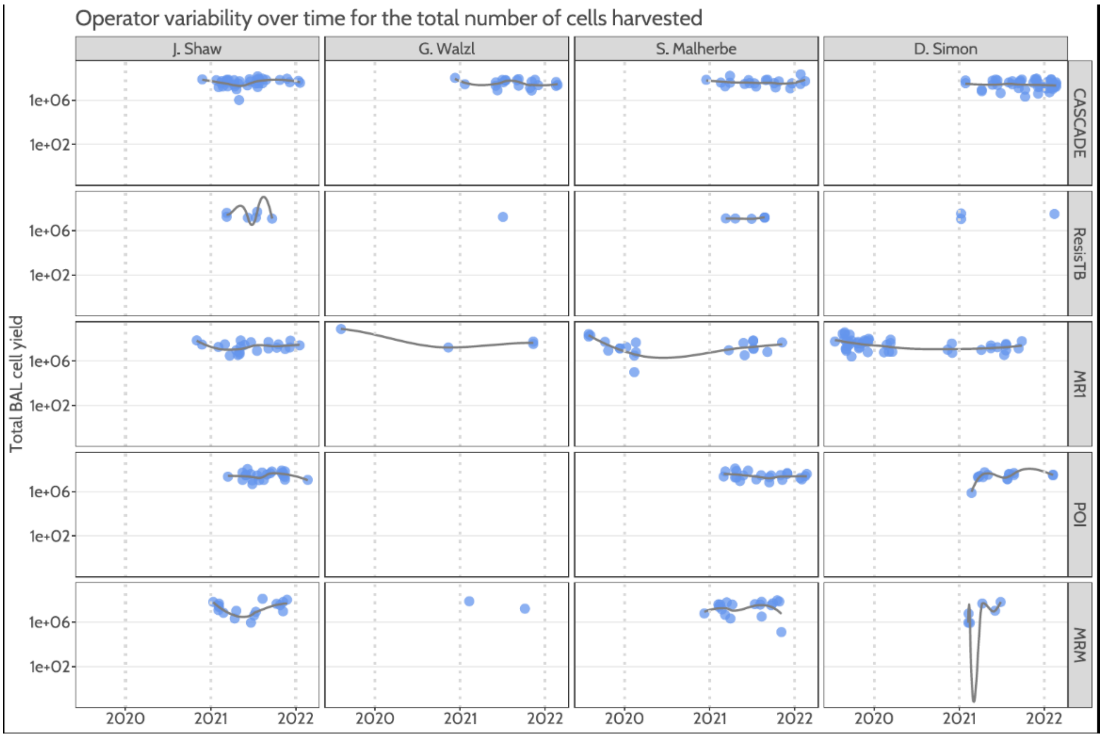

Panel C. There was no significant variability in the BAL cell yield over time when stratified by operator. Results were similar when analysed for each operator across all studies combined.

Supplementary Figure S2. The effects of BAL pellet colour on BAL yields.

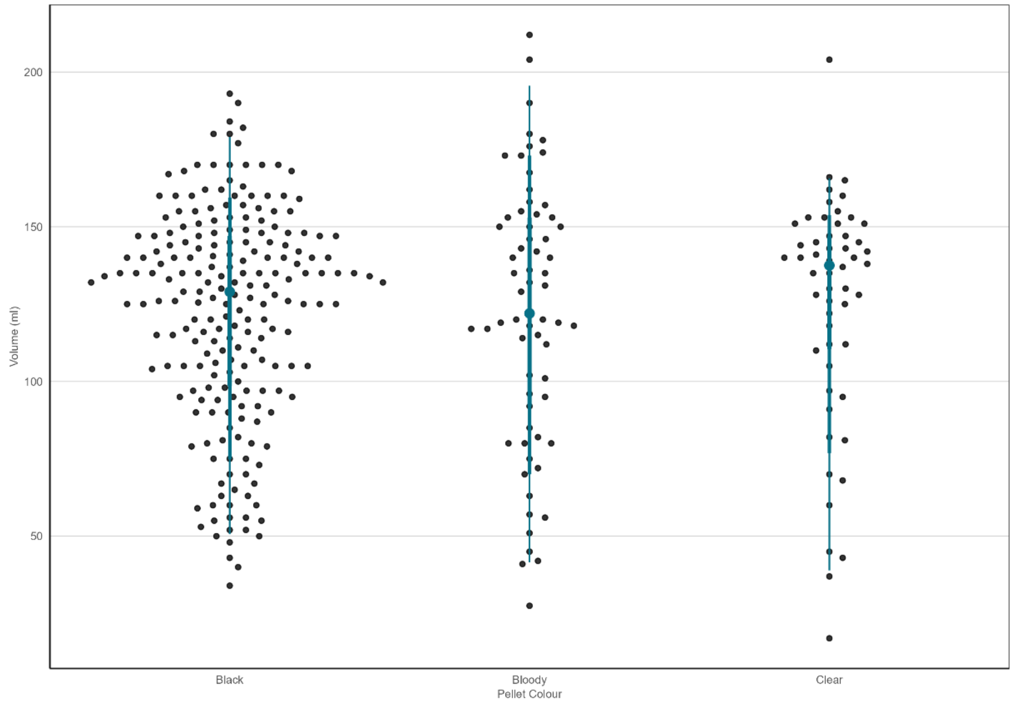

Panel A. The effect of pellet colour on BAL volume yield.

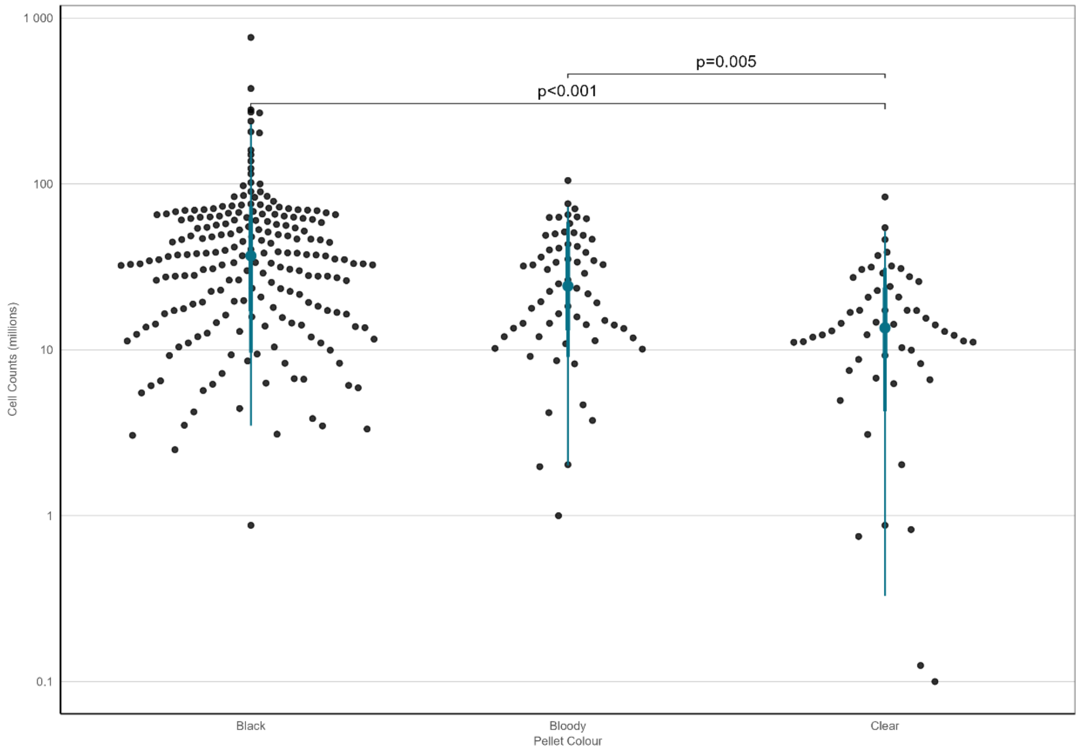

Panel B. The effect of pellet colour on BAL cell yield.

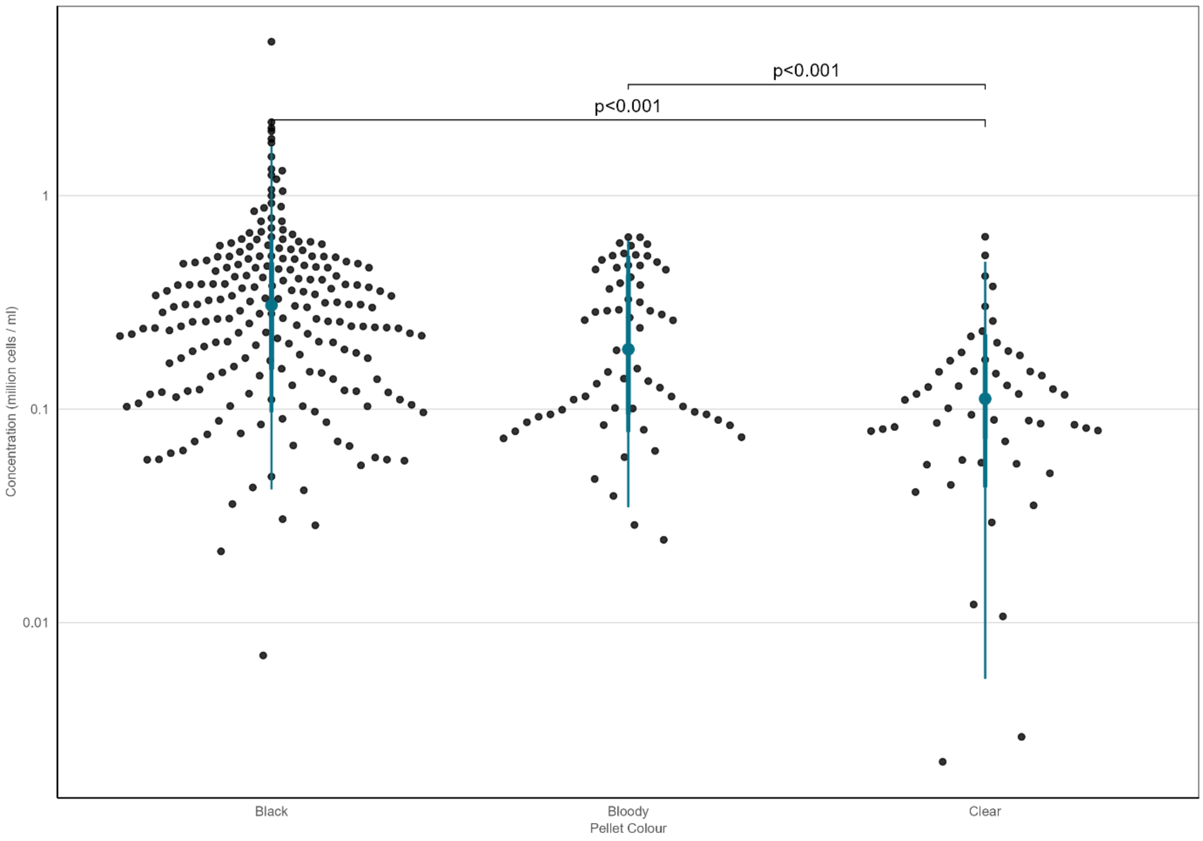

Panel C. The effect of pellet colour on BAL cell concentration.

Supplementary Figure S3. Bee swarm plots showing the effect of clinical group and TB treatment status on BAL volume, cell yield and cell concentration. (**A**) Volume yield; (**B**) Cell yield; (**C**) cell concentration. PDF, see Fig 3. ANOVA on medians: (**A**) F = 3.8874, P = 0.0015; (**B**) F = 4.7474, P = 0.001; (**C**) F = 4.2271, P = 0.0015.

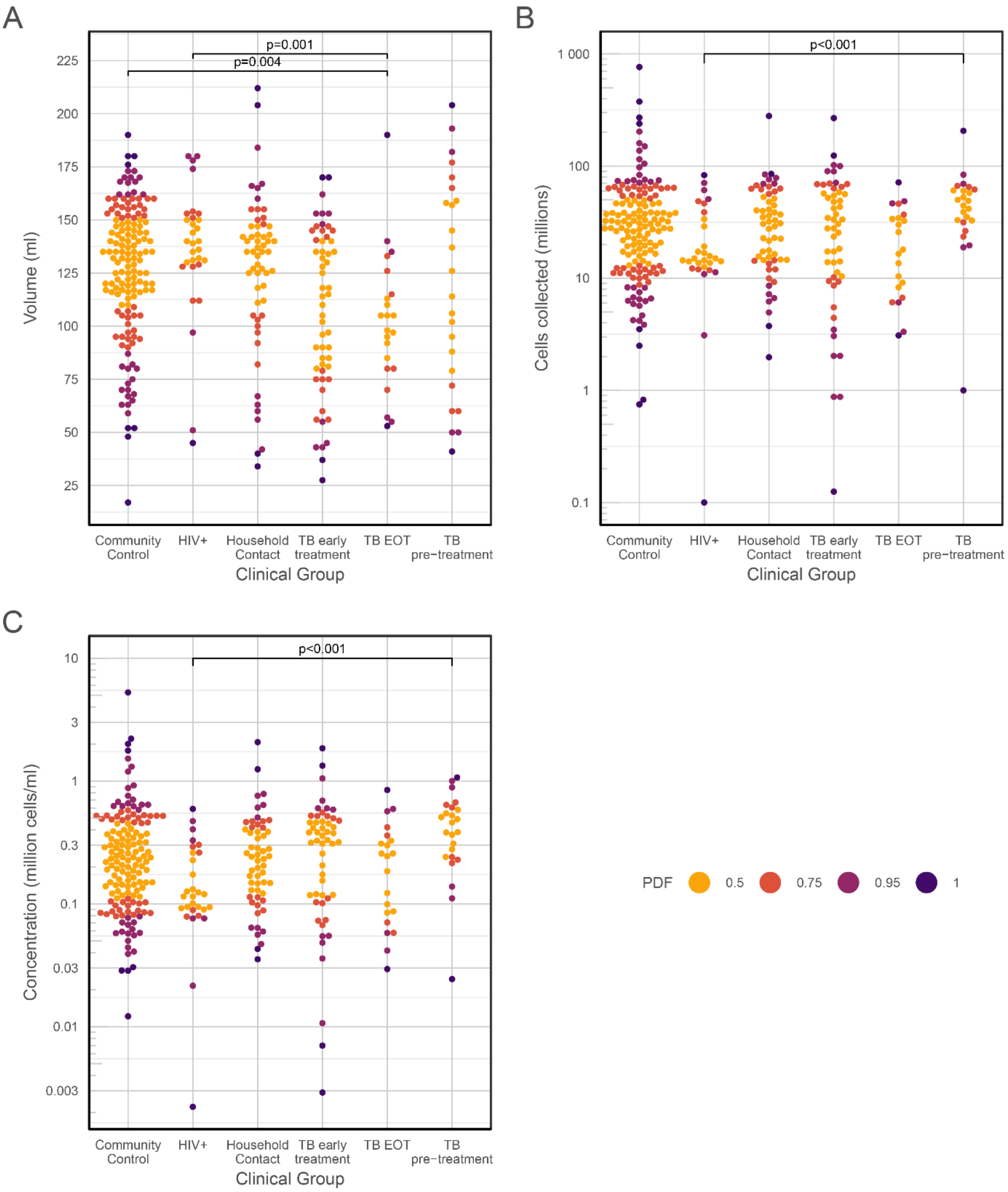

Supplementary Figure S4. The effect of bronchial brushings on BAL pellet colour. This time series shows the effect of performing a same segment bronchial brushing with a 'cytobrush' after the BAL compared to performing it in the same segment or in the ipsilateral lower lobe before the BAL. Dates in 'YY-mm' format (last two digits of year and a two-digit month). The Cochran-Armitage test for trends gave P < 0.001.

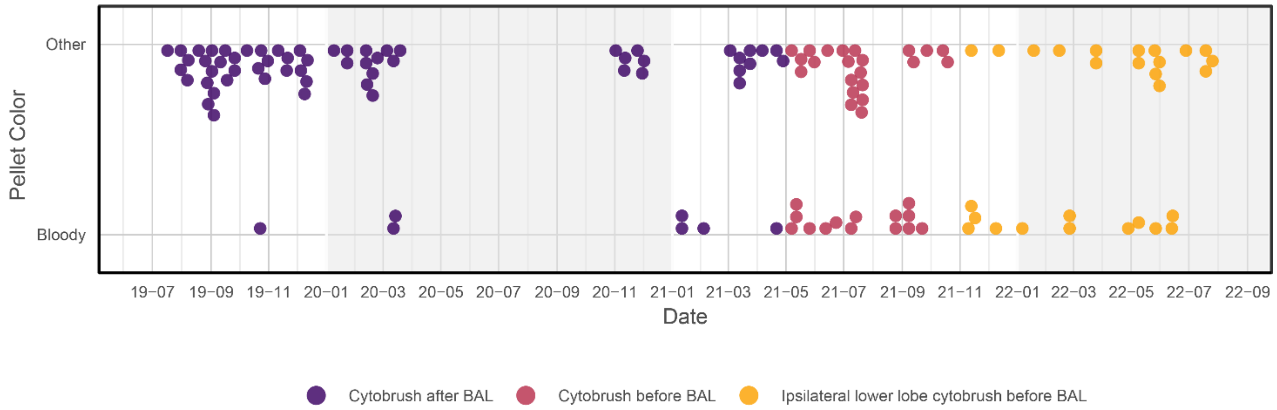

Supplementary Figure S5. Effects of (A) pellet colour, (B) age and (C) recent SARS-CoV-2 on BAL cell differential count.

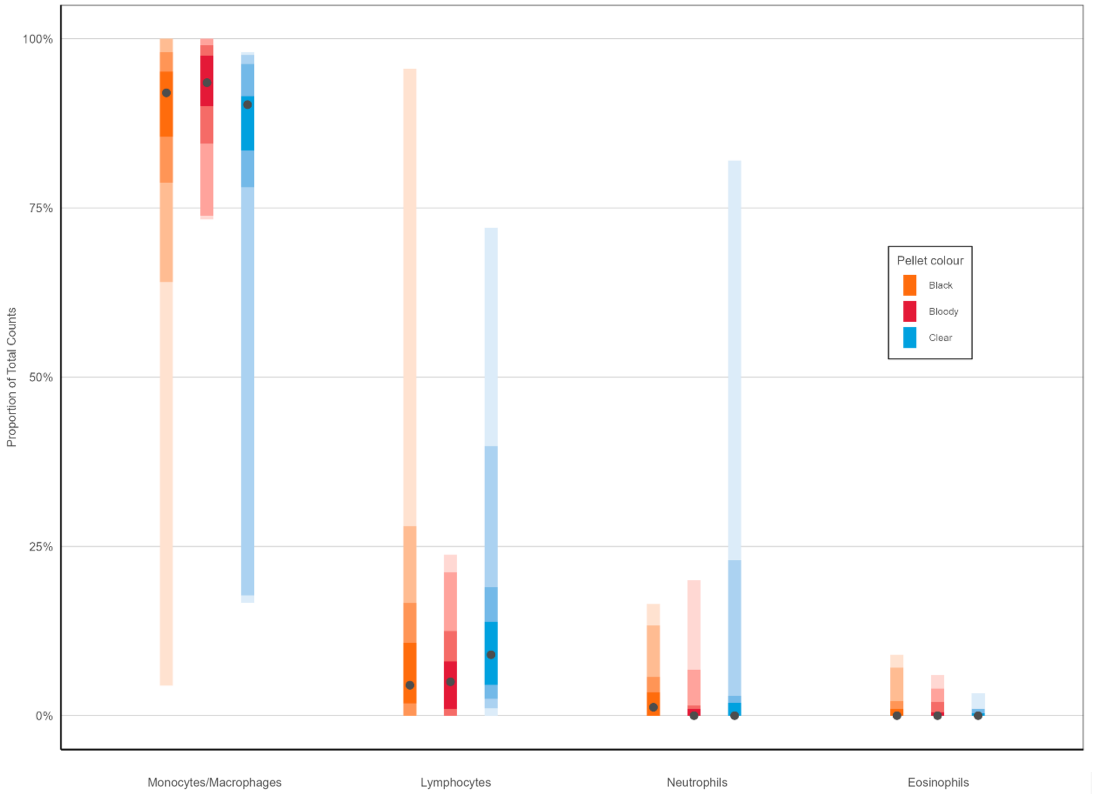

Panel A. Effects of pellet colour on BAL cell differential count. In panel (A), orange is black pellets; red is bloody pellets and blue is clear pellets. ANOVA on the means found that clear BAL pellets had the highest proportions of lymphocytes (P = 0.002), black pellets had the highest proportions of neutrophils (P = 0.01), and bloody pellets did not have any significantly enriched cell populations. P values have not been adjusted for multiple testing.

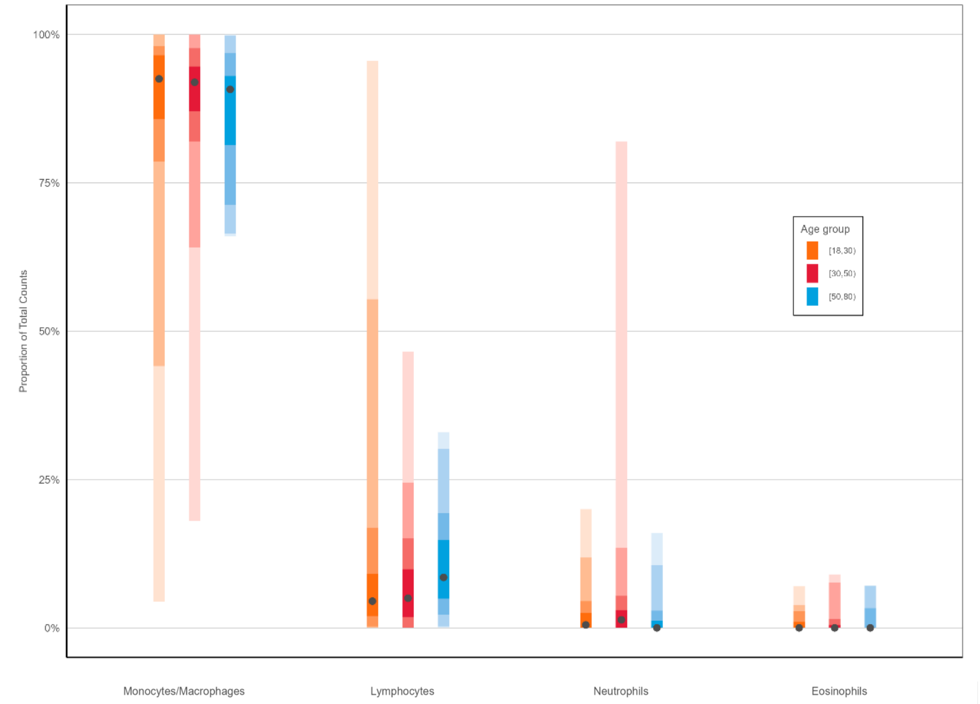

Panel B. The effects of age on BAL cell differential count. Age is considered in categories, where orange is 18-30; red is 30-50; and blue is over 50. ANOVA on the means was significant for differences in neutrophil counts (P = 0.003). P values have not been adjusted for multiple testing.

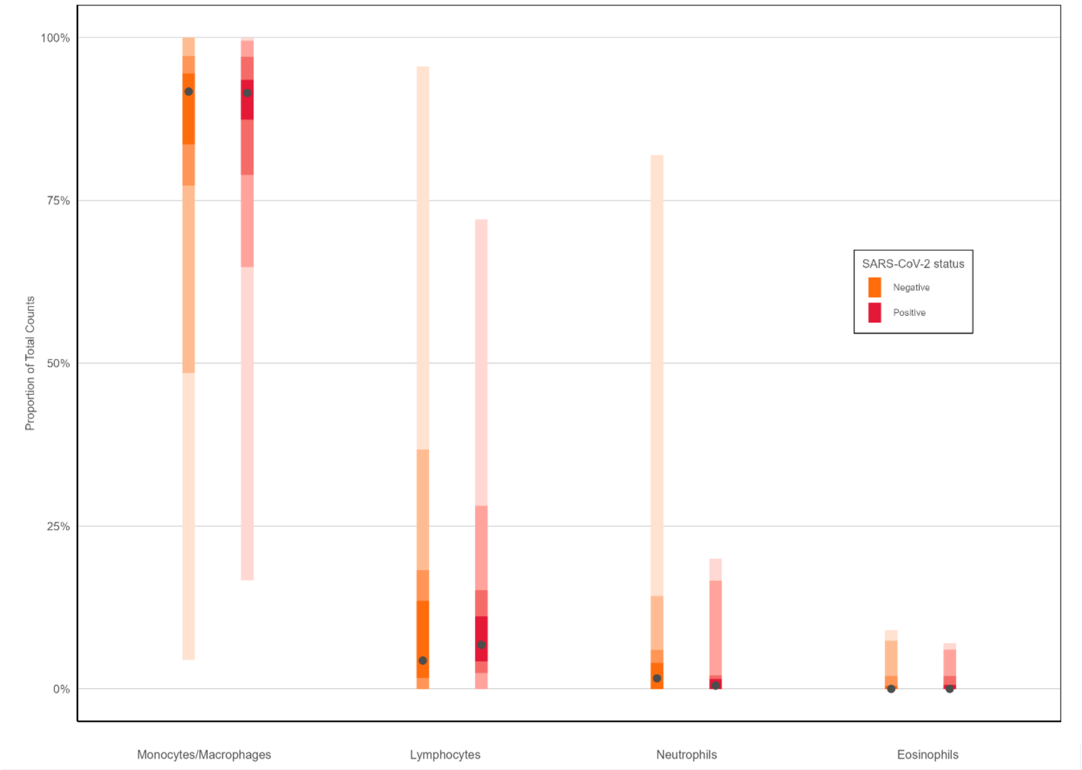

Panel C. The effect of recent SARS-CoV-2 infection on BAL cell differential counts. Orange is SARS-CoV-2 negative, and red is recent SARS-CoV-2 positive. ANOVA on the means found that SARS-CoV-2 positive participants had higher proportions of BAL lymphocytes than SARS-CoV-2 negative participants (P = 0.023). P values have not been adjusted for multiple testing.

Supplementary figure S6. Distribution of procedure times for procedures with and without brushings. The orange bars represent procedures with concomitant brushings and the pink bars represent procedures without concomitant brushings.

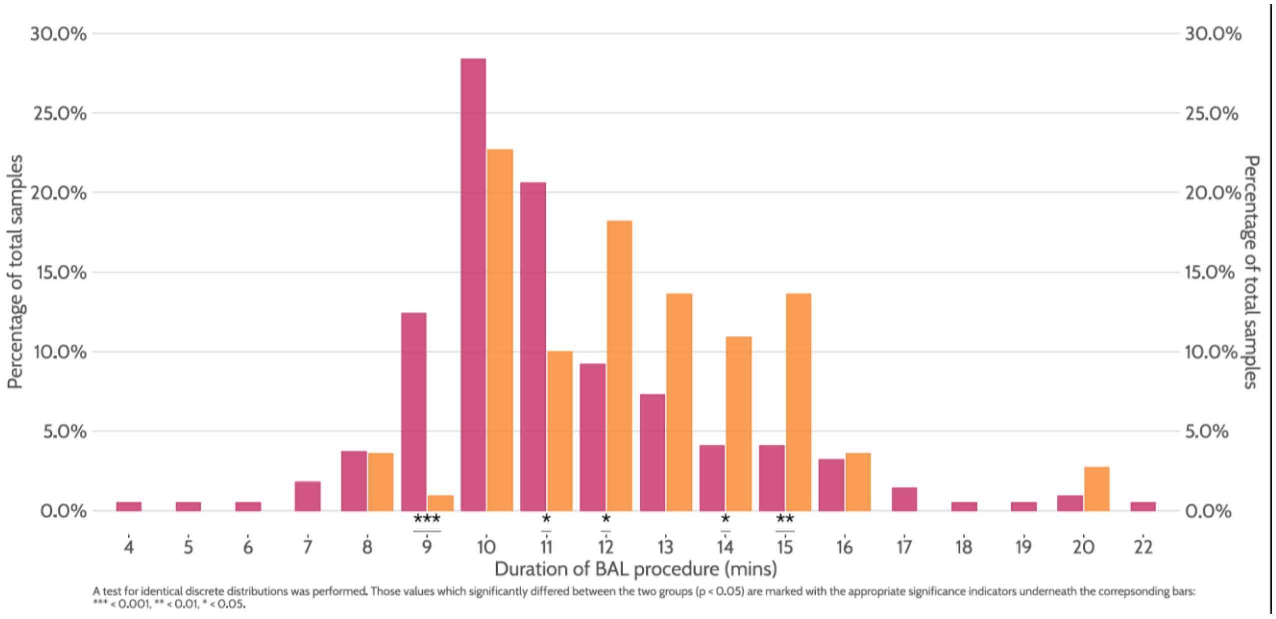

Supplementary figure S7. Minor adverse events during and immediately after the bronchoscopy with bronchoalveolar lavage, stratified by clinical group. The adverse events logged by study clinicians are noted in the rows, with event frequencies (percentage of participants who experienced this event) inside each cell. The colour scale provides an indication of the frequency. Grey cells are where no participants experienced this event. Columns denote the different clinical groups with the pooled values on the far left. Using a two-sided Cochran-Armitage test, a significant trend was found in hypertension in the first five clinical groups (excluding HIV+) (P = 0.034). Fisher's exact testing of HIV+ compared to the other groups found that minor bronchial mucosal bleeding was more common in this group (P < 0.001, 99.5% C.I. for odds ratio 1.9, 24.74).

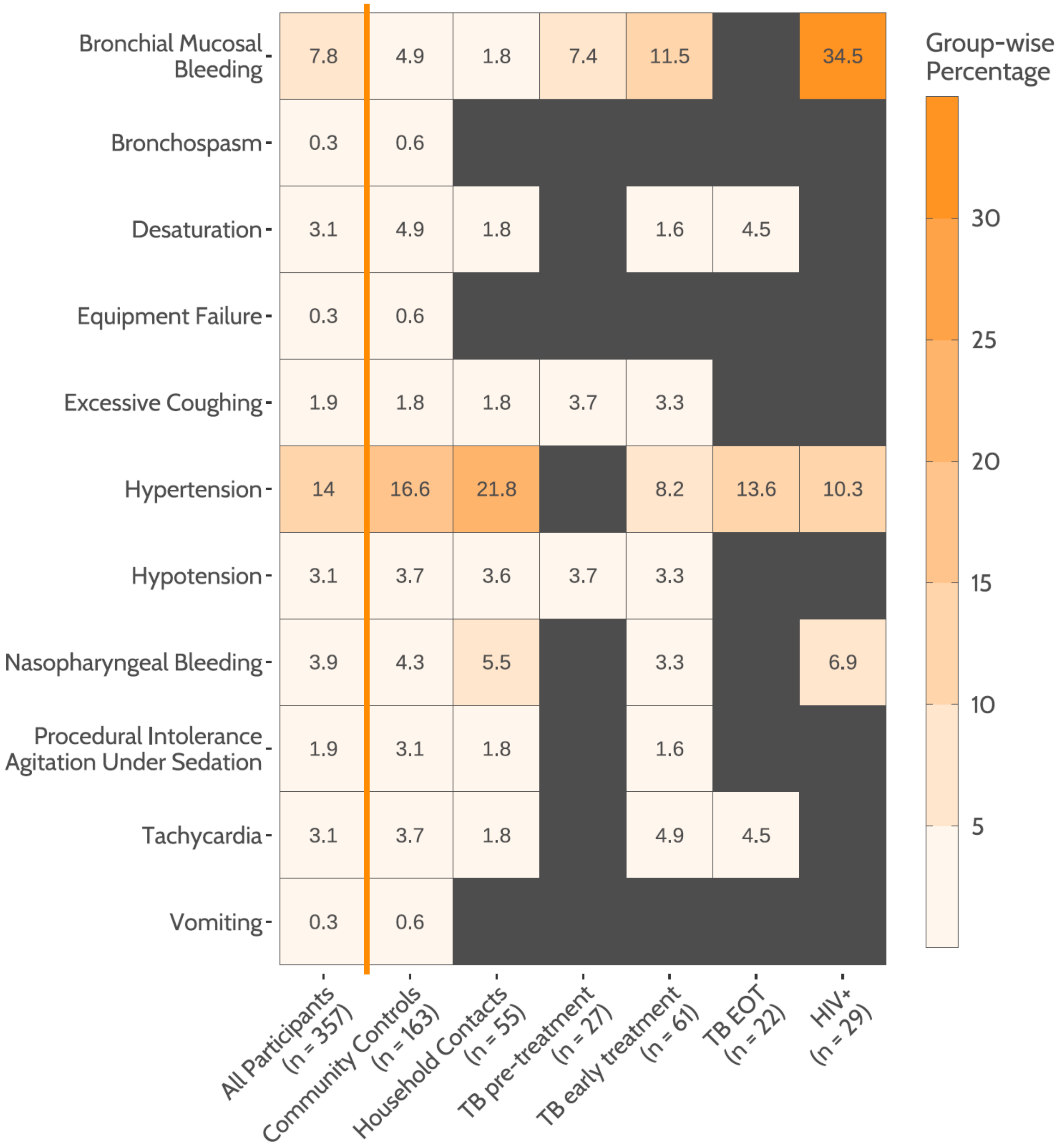

Supplementary Figure S8. Symptoms reported by participants 72 hours after bronchoscopy, stratified by clinical group. The symptoms reported by participants to study staff using standardised questionnaires at 72 hours after bronchoscopy are noted in the rows, with event frequencies (percentage of participants who experienced this event) inside each cell. The colour scale provides an indication of the frequency. Grey cells are where no participants experienced this event. Columns denote the different clinical groups with the pooled values on the far left. Using a two-sided Cochran-Armitage test, significant trends were found in 'throat discomfort' (P = 0.0264) and 'cough' (P = 0.044) in the first five clinical groups (excluding HIV+). Fisher's exact testing of HIV+ compared to the other groups found that dizziness was more common in this group (P = 0.010, 99.5% C.I. for odds ratio 0.48, 1199.94).

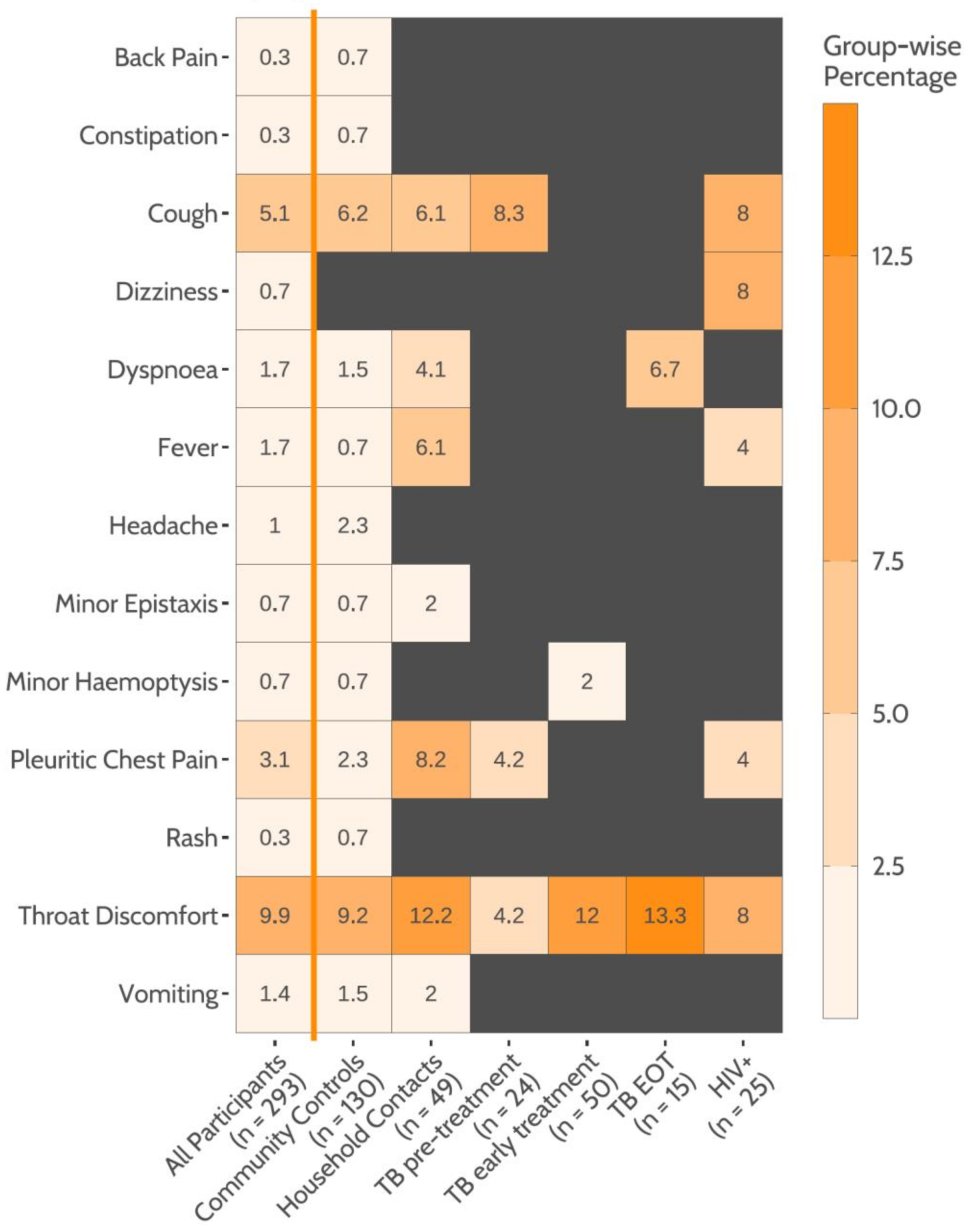

Supplement: Supplementary file 1 — Supplementary Information. [file 41598_2023_35723_MOESM1_ESM.pdf]
